# Mortality in a French military burn centre: a 12-year retrospective study analysing seasonal variations

**DOI:** 10.1186/cc14614

**Published:** 2015-03-16

**Authors:** M Boutonnet, C Dussault, N Donat, P Laitselart, A Cirodde, J Schaal, C Hoffmann, P Jault, T Leclerc

**Affiliations:** 1 HIA Percy, Clamart, France; 2 IRBA, Brétigny sur Orge, France

## Introduction

Factors associated with mortality in severely burned patients are well known. We aimed to assess the seasonal variations of the mortality of patients admitted to the main French military intensive care burn unit (ICBU) and to determine their relations to seasonal variations of severity or other factors (for example, staffing).

## Methods

We performed a retrospective study analysing the medical records of all patients admitted to the ICBU of Percy Military Hospital (France) between January 2000 and December 2011. Statistical analysis was performed with R version 3.1.2. We first conducted univariate analyses with simple logistic regression, then a simple periodic regression for seasonal variations (with and without harmonics for robustness), and finally a multivariate logistic regression with periodic terms in order to adjust seasonal variations for known severity factors.

## Results

A total of 1,913 patients were admitted for acute burn injury during the study period, with a male to female ratio of 2.34. Mean total body surface area burned (TBSA) was 23.2% (IQR: 10 to 30) and mean full thickness total body surface area burned (FTBSA) was 13.4% (IQR: 0 to 15). Inhalation injury was present in 441 (23.1%) cases, intoxication (CO, CN) was present in 88 (4.6%) cases, and associated traumatisms also in 88 (4.6%) cases. The mortality rate was 10.9%. The following factors were associated with mortality in univariate logistic regression: age, body mass index, past medical history, TBSA, FTBSA, intoxication (CO, CN), inhalation injury, flame burns, self-inflicted burns (all *P *< 0.0001), sex (*P *< 0.001), and admission date (*P *< 0.01). Simple periodic regression showed a biannual seasonal effect on mortality, documented with a 1-year periodic (*P *< 0.01) and a 6-month periodic (*P *= 0.01) dependency. Multivariate analysis with or without periodic terms identified age, past medical history, TBSA, FTBSA, inhalation, intoxication and admission date as the only factors independently associated with mortality.

## Conclusion

Predictive factors for mortality in our ICBU are in line with the literature. The documented seasonal variations in mortality are fully explained by variations in these severity factors. Complementary analyses are under way to further study a nonlinear age effect.

